# The Antidepressant Effect of Targeted Release of Ketamine-Loaded Nanodroplets Stimulated by Low-Intensity Focused Ultrasound

**DOI:** 10.3390/pharmaceutics17101251

**Published:** 2025-09-24

**Authors:** Bailing Wu, Yu Xu, Yuhang Xie, Youzhuo Li, Yue Huang, Yuran Feng, Mei Zhu

**Affiliations:** The First Affiliated Hospital of Kunming Medical University, Kunming 650032, China; wubailing@kmmu.edu.cn (B.W.);

**Keywords:** depression, ketamine, nanodroplets, low-intensity focused ultrasound (LIFU), lateral habenular

## Abstract

**Objectives**: Ketamine has demonstrated rapid and sustained antidepressant effects; however, its clinical utility is limited by the risk of addiction and systemic side effects. This study aimed to develop ketamine-loaded nanodroplets (Ket-NDs) with high encapsulation efficiency (EE) and stability for targeted low-dose intravenous (IV) administration in a mice model of depression. Low-intensity focused ultrasound (LIFU) was employed to induce transcranial, region-specific drug release in the lateral habenula (LHb). **Methods**: Ket-NDs were synthesized using a thin-film hydration method with sonication and emulsification, incorporating perfluoropentane as the core material. Characterization was performed using light microscopy, cryogenic scanning electron microscopy (cryo-SEM), transmission electron microscopy, and dynamic light scattering (DLS). Drug EE and loading efficiency (LE) were quantified by reversed-phase high-performance liquid chromatography. A chronic restraint stress model was established, and Ket-NDs were administered intravenously followed by LIFU targeting the LHb. Antidepressant efficacy and biosafety were systematically evaluated. **Results**: (1) Ket-NDs exhibited uniform spherical morphology and a narrow size distribution, as confirmed by DLS (particle size: 139.75 ± 9.43 nm; Polydispersity index: 0.225 ± 0.025) and cryo-SEM analysis (number-average diameter: 109.5 ± 10.4 nm). The zeta potential was −15.93 ± 5.906 mV, and the formulation remained stable under 4 °C storage. (2) Ket-NDs demonstrated high EE (78.25 ± 16.13%) and LE (15.55 ± 4.49%). (3) In depressive mice, IV administration of Ket-NDs followed by LIFU targeting the LHb significantly improved behavioral outcomes: increased locomotor activity in the open field test, elevated sucrose preference index, and reduced immobility time in the tail suspension test. (4) Safety assessments revealed no significant organ toxicity or brain tissue damage in ultrasound-exposed regions. **Conclusions**: In summary, this study developed stable Ket-NDs. When combined with LIFU, they enable precise regional drug delivery to the brain, showcasing a promising treatment strategy for depression with reduced systemic side effects.

## 1. Introduction

Depression is a prevalent chronic mental disorder. According to the World Health Organization (WHO), approximately 322 million people worldwide suffer from depression, accounting for 4.4% of the global population [1]. In 2008, the WHO ranked major depressive disorder as the third leading cause of global disease burden, with projections suggesting it may surpass cardiovascular diseases and cancer to become the primary disease burden by 2030 [2]. Current clinical treatments primarily include psychotherapy, pharmacotherapy, and physical therapy. However, depression has a complex etiology. This, coupled with the diversity and heterogeneity of patient symptoms, unpredictable prognosis, and varied treatment responses, often leads to suboptimal therapeutic outcomes. Furthermore, it contributes to high relapse rates after medication discontinuation. Notably, 40–50% of patients fail to achieve symptomatic remission despite receiving treatment [3], imposing substantial economic and psychological burdens on both society and affected families.

The rapid antidepressant effects of ketamine (Ket) have been recognized as ‘the most significant discovery in the field of mental illness in the past half-century’ [4]. Administration of Ket induces rapid and sustained antidepressant [5,6] and anti-suicidal effects [7]. For patients with treatment-resistant depression, even a single subanesthetic dose of Ket can rapidly alleviate depressive symptoms [8,9]. A seminal study by Prof. Hailan Hu’s team [10] elucidated its core neural mechanisms: under depressive states, the expression of Kir4.1 channels in astrocytes of the lateral habenula (LHb) increases, leading to reduced extracellular potassium concentration around neuronal somata, membrane hyperpolarization, and subsequent activation of T-type voltage-sensitive calcium channels (T-VSCC). This triggers N-methyl-D-aspartate (NMDA) receptor-dependent ‘burst firing.’ This activity potently suppresses downstream reward centers, thereby inducing depressive behaviors. Importantly, Ket rapidly alleviates depression by blocking this NMDA-mediated burst firing in the LHb [4,11]. However, its clinical utility is constrained by its hallucinogenic and addictive side effects [12], necessitating low-dose strategies to minimize systemic exposure.

Current brain-targeted delivery technologies, such as intracranial injection [13], implantable devices [14], and convection-enhanced delivery (CED) [15], can achieve high local drug concentrations in brain tissue. However, these methods risk brain injury and infection, limiting repeated treatments and multi-site administration. Intranasal drug delivery provides a non-invasive alternative for drug administration. This approach transports drugs from the submucosal space to the brain parenchyma through the cerebrospinal fluid (CSF) pathway. However, it is limited by slow cerebrospinal fluid distribution and uneven drug dispersion [16].

With the advancement of nanotechnology, the integration of nanomaterials and medicine has deepened, leading to rapid developments in nano-drug delivery systems (NDDSs). By optimizing the design and functionalization of nanocarriers, NDDS enhances drug solubility and stability while improving bioavailability. These systems ensure structural integrity during drug delivery and enable targeted transport and controlled release, thereby increasing therapeutic efficacy and reducing adverse effects [17]. Among various nanoplatforms, nanodroplets (NDs) have emerged as a research focus in recent years due to their significant potential in drug delivery applications. NDs are submicron-scale (100–400 nm) liquid perfluorocarbon (PFC) particles encapsulated by phospholipid or polymeric shells for stability [18]. The metastable PFC core undergoes acoustic droplet vaporization (ADV) upon ultrasound stimulation, transforming into microbubbles (MBs) that combine the deep tissue penetration of nanoparticles (in liquid state) with the cavitation effects of MBs (after vaporization) [19]. As such, NDs have been widely applied in brain tumor therapy [20], treatment of other brain-related disorders, blood–brain barrier (BBB) opening [21,22], and neuromodulation [23].

Ultrasound, characterized by its cavitation, mechanical, and thermal effects, represents an efficient exogenous stimulus for controlled drug release. Transcranial ultrasound coupled with nanoemulsions has been found to enable efficient, low-power drug targeting in neurological models [24]. Under ultrasound stimulation, the liquid core of NDs undergoes phase transition into a gaseous state, resulting in volume expansion and vaporization into MBs. Ultrasonic oscillations perturb fluid flow around MBs, generating cavitation microstreaming that influences surrounding blood cells or vascular walls [25]. This process induces phospholipid membrane disruption in adjacent cells, forming sonopores that enhance blood perfusion and promote molecular drug uptake [26]. With increasing acoustic intensity, the kinetic behavior of cavitation nuclei becomes more violent under acoustic pressure, exhibiting nonlinear oscillation characteristics. During the negative-pressure phase, cavitation bubbles rapidly expand, followed by violent contraction and eventual implosion during the positive-pressure phase [27], creating controllable cavitation effects within the sonication region to improve drug permeability [28].

Our previous work further revealed that transcranial low-intensity focused ultrasound (LIFU) combined with MBs alleviates symptoms in heroin-addicted mice [29]. Compared to conventional MBs, NDs exhibit several distinct advantages, making them a promising alternative for therapeutic applications. MBs typically range from 1 to 10 μm in diameter, a size that promotes rapid recognition and clearance by the reticuloendothelial system (RES), resulting in most being phagocytosed and eliminated within minutes after intravenous injection [30]. In contrast, the substantially smaller size of NDs not only delays RES-mediated clearance—enabling an extended circulation half-life of up to 20–40 min depending on formulation [31]—but also facilitates penetration through vascular endothelial gaps into tumor interstitium, overcoming the intravascular limitation typical of MBs [32]. Moreover, under high acoustic pressure, MBs are susceptible to inertial cavitation, which can cause violent bubble collapse, microjet formation, and subsequent vascular damage, erythrocyte extravasation, or edema. NDs, however, undergo acoustic droplet vaporization to form uniformly sized MBs that predominantly exhibit stable cavitation, thereby reducing the risk of vascular injury [33]. Additionally, while conventional MB preparations are often polydisperse, leading to inconsistent acoustic responses and heterogeneous drug delivery [34], NDs can be engineered with modified shells to enhance in vivo stability and biocompatibility [35], offering more predictable behavior and improved therapeutic uniformity. Recent evidence indicates that loading Alzheimer’s disease therapeutics into lipid nanoemulsions significantly reduces the required power for transcranial ultrasound therapy, enabling safe and efficient drug delivery to specific brain regions [36]. There are also studies that use focused ultrasound combined with GDNF-loaded MBs to open the BBB and release it in a targeted manner to treat depression in rats [37].

**Figure 1:**
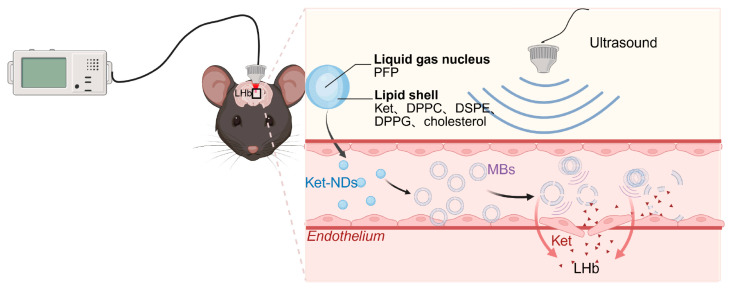
Schematic illustration of ultrasound-triggered drug release from Ket-NDs. Upon irradiation with sufficient acoustic energy, the liquid perfluorocarbon core of Ket-NDs undergoes phase transition through ADV, facilitated by combined cavitation and thermal effects, transforming into MBs. Under sustained ultrasound exposure, the MBs induce cavitation-mediated BBB disruption. Concurrently, the released Ket permeates through the opened BBB into the targeted brain region. Created with BioRender.com.

Therefore, this study aimed to develop ketamine-loaded nanodroplets (Ket-NDs) with high encapsulation efficiency (EE) and stable physicochemical properties. Following intravenous administration, transcranial LIFU was applied to precisely irradiate target brain regions. This combinatorial therapeutic strategy achieves dual objectives. (1) Ultrasound-triggered cavitation transiently opens the blood–brain barrier, and (2) spatially controlled vaporization releases Ket specifically within the LHb, suppressing pathological burst firing while mitigating dose-limiting adverse effects (Figure 1). The Ket-NDs were synthesized and characterized for critical quality attributes including particle size distribution, zeta potential, EE and loading efficiency (LE). An established chronic restraint stress model was induced in C57BL/6 mice through a 14-day restraint stress protocol (4 h/day). Ket-NDs were intravenously administered to depressed mice, followed by transcranial LIFU irradiation of the LHb to trigger targeted drug release. Antidepressant-like effects were quantitatively assessed through a standardized behavioral test battery including the sucrose preference test (SPT) for anhedonia, open field test (OFT) for locomotor activity and anxiety-like behavior, forced swim test (FST) and tail suspension test (TST) for behavioral despair. Additionally, histological analyses were performed to evaluate treatment safety.

## 2. Materials and Methods

### 2.1. Materials

For Ket-ND preparation, 1,2-dipalmitoyl-sn-glycero-3-phosphatidylcholine (DPPC, Cat#: LP-R4-057, ≥99.0%, Molecular Weight (MW): 733 g/mol), 1,2-distearoyl-sn-glycero-3-phosphoethanolamine (DSPE, Cat#: LP-R4-020, ≥99.0%, MW: 748 g/mol), 1,2-dipalmitoyl-sn-glycero-3-phospho-(1′-rac-glycerol) (DPPG, Cat#: LP-R4-016, ≥99.0%, MW: 744 g/mol), and cholesterol (Cat#: R-H-100001, ≥99.0%, MW: 386.65 g/mol) were purchased from Xi’an Ruixi Biotechnology Co., Ltd. (Xi’an, China). Chloroform (CAS: 67-66-3, ≥99.0%, stabilizer: 0.3–1.0% ethanol, MW: 119.38 g/mol) was purchased from Sichuan Xilong Science Co., Ltd. (Chengdu, China). Perfluoropentane (PFP, CAS: 678-26-2, Cat#: STREM-09-6182, ≥98.0%, MW: 288.03 g/mol) was purchased from Strem Chemicals, Inc. (Newburyport, MA, USA). Ketamine hydrochloride (CAS: 1867-66-9, 99.8% purity by HPLC, MW: 274.19 g/mol) was purchased from Shanghai Yuansi Standard Technology Co., Ltd. (Shanghai, China). Phosphate buffered saline (PBS) was purchased from Servicebio Technology Co., Ltd. (Wuhan, China).

For in vivo studies, normal saline (NS, 0.9% sodium chloride), hematoxylin and eosin (H&E) staining kit were purchased from Servicebio Technology Co., Ltd. (Wuhan, China). Sucrose was purchased from Solarbio Science & Technology Co., Ltd. (Beijing, China). Isoflurane was purchased from RWD Life Science Co., Ltd. (Shenzhen, China). Pentobarbital sodium was purchased from Shanghai Macklin Biochemical Technology Co., Ltd. (Shanghai, China).

### 2.2. Preparation of Ket-NDs

A precisely weighed fixed-ratio mixture of DPPC, DSPE, DPPG, and cholesterol was dissolved in chloroform in a round-bottom flask and incubated at 50 °C in a water bath until complete lipid dissolution. Ketamine hydrochloride was dissolved in PBS to prepare a 1.5 mg/mL Ket solution. The Ket solution was added to the round-bottom flask, and the mixture was subjected to rotary evaporation (80 rpm, 50 °C) until a thin lipid film formed with no residual solvent. The film was then rehydrated with PBS under gentle agitation.

Under continuous ice-bath conditions, the lipid suspension was emulsified using an ultrasonic cell disruptor (Yuanshengte Intelligent Technology Co., Wuxi, Jiangsu, China) with the probe tip positioned precisely at the liquid–liquid interface of the lipid solution. The instrument was operated at 100 W with intermittent pulses (5 s on/5 s off, 50% duty cycle) for 6 min. Subsequently, PFP was added dropwise, and the mixture was further sonicated at the liquid–liquid interface to form a milky suspension. The suspension was centrifuged (3300× *g*, 4 °C, 5 min) using a high-speed centrifuge (Xiangyi, China; model H1850R with No. 2 angle rotor) to remove unencapsulated material, and the supernatant was discarded. This washing step was repeated three times to purify the Ket-NDs. The resulting pellet was resuspended in PBS and subsequently passed through a Millex-GP syringe filter unit (0.22 μm pore size; Merck Millipore, Darmstadt, Hesse, Germany) for sterilization and removal of large aggregates. The purified Ket-NDs were finally stored at 4 °C for further experiments.

### 2.3. Physical Characteristics and Characterization of Ket-NDs

The morphology, particle size distribution, and homogeneity of Ket-NDs were initially examined under an optical microscope (IX72, Olympus Corp., Tokyo, Japan) at 200–400× magnification, with particle density quantified using a hemocytometer (Marienfeld Superior, Lauda-Königshofen, Baden-Württemberg, Germany). Key physicochemical parameters including average size distribution, polydispersity index (PDI) and zeta potential were determined using a nanoparticle and zeta potential analyzer (Brookhaven Instruments Corporation, Holtsville, NY, USA). All measurements were performed in triplicate to ensure data reliability.

For detailed structural analysis, the Ket-NDs were characterized by cryogenic scanning electron microscopy (Cryo-SEM; HITACHI SU8200, Tokyo, Japan) and transmission electron microscopy (TEM; Jeol JEM-1400Flash, Tokyo, Japan).

#### 2.3.1. Cryo-SEM

Ket-ND samples were mounted on cryo-stubs to form mushroom-shaped tops, cryo-fixed by rapid immersion in supercooled liquid nitrogen slush (−210 °C) under 0.1 Pa vacuum, transferred via a Quorum PP3010T system to a precooled stage (−140 °C), fractured with a pre-cooled blade, freeze-dried at −90 °C for 30 min, sputter-coated with Pt at −140 °C for 60 s with 9 mA current, and imaged on the Cryo-SEM system at 1.0 kV and 11.2 mm working distance in mixed SE mode (anti-contamination traps: <−170 °C; magnification: 3000–50,000×).

#### 2.3.2. TEM

Homogenized samples were randomly aspirated in duplicate (10 μL per aliquot) and deposited onto clean carbon-coated copper grids (300 mesh). Negative staining was performed using 2% phosphotungstic acid as the heavy metal stain. After 30 s staining at pH 7.0, grids were air-dried at room temperature. TEM observation was conducted on the aforementioned TEM instrument operating at 100 kV (magnification: 40,000–80,000×).

Particle size analysis was performed on a representative Cryo-SEM image using Image-J software (version 1.46r, National Institutes of Health, Bethesda, MD, USA) for particle sizing and data recording. The image was spatially calibrated against its embedded scale bar at a magnification of 30,000×. Individual particles were manually identified and outlined using the freehand selection tool to ensure accurate delineation of boundaries. The area of each traced particle was recorded. The equivalent circular diameter was then calculated from the measured area using the software’s built-in functions. To ensure a representative and statistically robust analysis, over 200 discrete, well-dispersed particles were measured. Subsequent statistical analysis was conducted using GraphPad Prism 10 (GraphPad Software, San Diego, CA, USA). The frequency distribution of the diameters was plotted and fitted to a Gaussian model. Final size distributions were presented as frequency histograms with 20 nm bin intervals. Mean diameter ± standard deviation (SD) and percentile values (D_10_, D_50_, D_90_) were calculated to characterize population heterogeneity.

### 2.4. Determination of EE and LE by Reversed-Phase High-Performance Liquid Chromatography (RP-HPLC)

Chromatographic conditions: The analysis was performed using an Agilent ZORBAX SB-C18 column (4.6 mm × 250 mm, 5 μm) with a mobile phase consisting of acetonitrile−0.4% phosphoric acid aqueous solution (12: 88, *v*/*v*) at a flow rate of 1 mL/min. The column temperature was maintained at 25 °C, with an injection volume of 10 μL and detection wavelength set at 220 nm [38].

Preparation of stock solution: Ketamine hydrochloride standards (10.22 mg) were accurately weighed and dissolved in mobile phase, then diluted to 10 mL with purified water to obtain a stock solution of 1.022 mg/mL.

Calibration curve: The stock solution was diluted to prepare standard solutions at concentrations of 0.10217, 0.5108, and 1.022 mg/mL. Aliquots (10 μL) of each standard solution were injected for RP-HPLC analysis. A calibration curve was constructed by plotting peak area (*Y*-axis) versus Ket concentration (*X*-axis).

Sample analysis: The post-centrifugation supernatant (containing unencapsulated drug) collected during Ket-ND preparation was analyzed using an Agilent 1100 HPLC system (Agilent Technologies, Santa Clara, CA, USA). The drug concentration in the supernatant was determined using the calibration curve, and EE and LE were calculated as follows:

$$The\;Ket\;EE\;(\%)=\frac{total\;Ket-unloaded\;Ket}{total\;Ket}\times 100\%$$

$$The\;Ket\;LE\;(\%)=\frac{total\;Ket-unloaded\;Ket}{Total\;phospholipids}\times 100\%$$

### 2.5. Stability Assessment of Ket-NDs

At the 0, 24, and 48 h time points under 4 °C storage, Ket-ND samples were taken, and their particle size was detected using a nanoparticle and zeta potential analyzer. To ensure accuracy, triplicate measurements were performed at each time point.

Under 4 °C storage conditions, samples of Ket-NDs were collected at fixed time intervals, and the concentration of free Ket was quantified using the RP-HPLC method described in Section 2.4, with the cumulative drug release rate calculated according to the following formula:

$$cumulative\;drug\;release\;rate\;(\%)=\frac{free\;Ket}{total\;Ket-unloaded\;Ket}\times 100\%$$

### 2.6. In Vitro Release of Ket-NDs Under Ultrasound Irradiation

The LIFU system (developed collaboratively with SIAT-CAS) was used to irradiate the Ket-NDs at room temperature. The sonication parameters were as follows: frequency: 2 MHz; duty cycle: 5%; pulse duration: 500 μs; pulse repetition period: 1 ms; stimulus duration: 300 ms; inter-stimulus interval: 3 s; peak negative pressure (PNP): 3.08 MPa. A control group was kept at room temperature without ultrasound exposure. Aliquots of Ket-NDs were collected at predetermined time intervals (0, 5, 10, 15, and 20 min), and the amount of cumulative released Ket was determined via RP-HPLC. The cumulative drug release rate was calculated as described in Section 2.6, and a release profile was plotted as a function of time.

### 2.7. Experimental Paradigm

**Figure 2:**
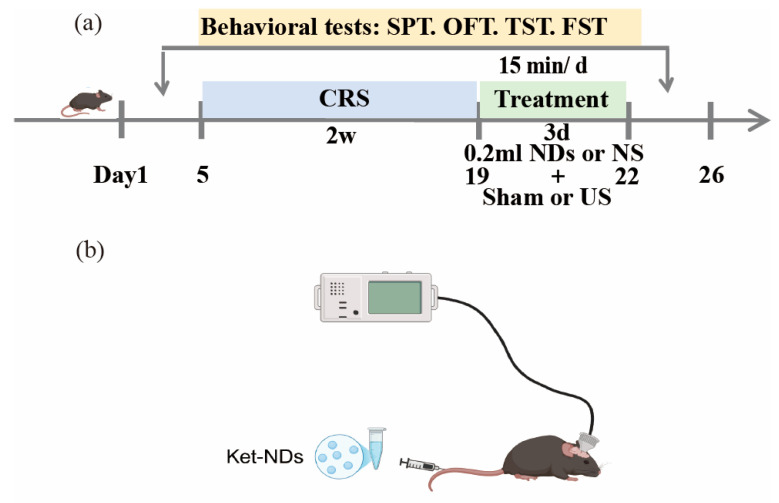
Experimental paradigm. (**a**) Timeline of the experimental procedure. (**b**) Schematic depiction of the experimental methods: The head-mounted ultrasound probe which is attached to the acoustic generator was snugly placed against the scalp surface corresponding to the LHb brain region in mice. Created with BioRender.com.

The experimental design is illustrated in Figure 2. Prior to baseline behavioral testing, newly acquired mice were acclimatized to housing and experimental conditions for 7 days with ad libitum access to food and water, followed by body weight measurement. After 1 week of adaptive feeding, the mice were randomly divided into a control group, chronic restraint stress (CRS) group, CRS + Ket-NDs + Sham group, CRS + NS + US group, CRS + Ket-NDs + US group. Baseline behavioral assessments were performed on all animals, including SPT, OFT, TST and FST. Subsequently, mice in groups CRS, CRS + Ket-NDs + Sham, CRS + NS + US, and CRS + Ket-NDs + US were subjected to CRS for 4 h daily over 14 consecutive days. The CRS + NS + US and CRS + Ket-NDs + US group received transcranial LIFU irradiation for 3 consecutive days, while sham irradiation was performed for other groups. During ultrasound sessions, both the CRS + Ket-NDs + US and CRS + Ket-NDs + Sham groups were intravenously administered 0.2 mL Ket-NDs via tail vein injection, whereas the remaining groups received 0.2 mL NS. Post-treatment behavioral evaluations (SPT, OFT, TST, FST) were performed to assess antidepressant-like effects. Following euthanasia, major organs (heart, liver, spleen, lungs, and kidneys) and brain tissues were harvested. Histopathological evaluation was performed using H&E staining to assess treatment safety.

### 2.8. Animals

Male C57BL/6 mice (6–8 weeks old, 20 ± 2 g) were obtained from the Experimental Animal Center of Yunnan University (Yunnan, China) and housed in the animal facility of Kunming Medical University. Mice were raised under standard conditions in individually ventilated cages, and mice could move freely with sufficient food and water. A 12 h light–dark cycle (lights on at 07:00, off at 19:00) was maintained throughout the study. This study was approved by the Animal Ethics Committee of Kunming Medical University (Approval No. kmmu20211403, Approval No. kmmu20241609), adhering to principles of replacement, reduction and refinement (3Rs). Surgical procedures were performed under appropriate anesthesia and analgesia to minimize animal suffering.

### 2.9. Procedure of CRS

**Figure 3:**
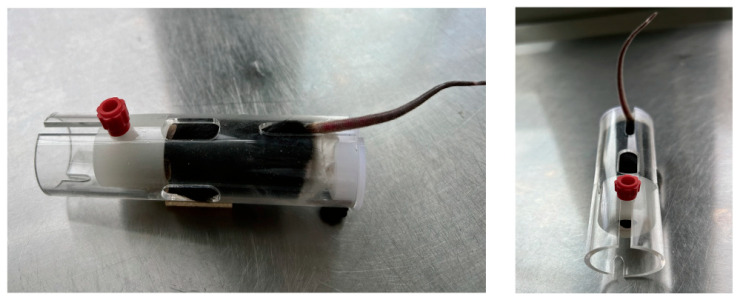
Transparent plastic cylindrical restraint tubes for chronic restraint stress in mice.

The CRS model was established following a validated protocol [39]. Specifically, mice were individually restrained in ventilated transparent plastic tubes (25 mm diameter × 110 mm length) for 4 h daily over 14 consecutive days (Figure 3). Adjustable plastic inserts were positioned to restrict movement without causing injury, allowing limited head and limb mobility while preventing body rotation. During restraint sessions, mice were deprived of food and water.

### 2.10. Behavioral Assessments

Following the 14-day restraint period, mice underwent a standardized behavioral test battery including SPT, OFT, TST and FST. All tests were performed during the dark (active) phase of the 12 h light/dark cycle. Mice were acclimated to the testing room for 3 h prior to experiments, which were conducted under dim lighting and low-noise conditions. Behavioral sessions were recorded using a video tracking system (Any-maze, Stoelting Co., Wood Dale, IL, USA) and analyzed by investigators blinded to experimental conditions.

SPT:

Prior to testing, mice were habituated to two bottles of purified water for 24 h followed by 24 h exposure to two bottles of 1% sucrose solution. After 24 h food and water deprivation, mice were individually presented with one bottle of 1% sucrose solution and one bottle of purified water during the 12 h dark cycle (21:00–09:00). Fluid consumption was measured, and sucrose preference was calculated as (sucrose intake)/(total fluid intake) × 100% [40].

OFT:

Mice were gently introduced along the wall of a 50 cm × 50 cm × 40 cm (length × width × height) open field arena, which was virtually divided into 16 virtual squares (12.5 cm × 12.5 cm each). The central four quadrants were designated as the center zone by automated tracking software. Mice were allowed to move inside the box freely. Following a 1 min habituation period, locomotor activity was recorded for 10 min using an overhead video tracking system. Throughout testing, environmental conditions were maintained at minimal noise levels. Between trials, the arena was meticulously sanitized with 75% ethanol to eliminate residual odor cues that might influence subsequent behavioral measurements. The total moving distance, immobility time and mean speed were used to assess locomotor activity [4].

TST:

Mice were suspended by adhesive tape placed 1–2 cm from the tail tip on a horizontal bar (head 10–15 cm above the surface). Behavior was recorded for 6 min, with immobility time during the last 4 min quantified by automated analysis.

FST:

Mice were placed individually in a transparent cylinder (12 cm diameter × 25 cm height) filled with 15 cm depth water (23–25 °C) and the swimming animal could not touch the bottom of the container with their posterior limbs or tails. After 6 min swimming sessions, immobility time (defined as passive floating with only movements necessary to maintain balance) during the final 4 min was analyzed using side-view camera recordings [41].

### 2.11. Targeted Release of Ket-NDs via Transcranial LIFU Irradiation

The LIFU system was developed by the Shenzhen Institutes of Advanced Technology, Chinese Academy of Sciences (SIAT-CAS), comprising a high-voltage waveform generator, matching circuits, and switch-mode power amplifier [36]. The focused ultrasound transducer (composite single-crystal material, 20 mm outer diameter, 19 mm radius of curvature) operated at a fundamental frequency of 2 MHz. The transducer was mounted on a custom collimator filled with degassed water and sealed with parafilm, then coupled to the mice scalp using ultrasound gel.

Acoustic field measurements were performed using an automated 3D acoustic scanning system (UMS3, Precision Acoustics, Dorchester, UK). The setup consisted of a control PC, a signal acquisition oscilloscope, a 3D positioning stage, and a hydrophone. A needle hydrophone (Precision Acoustics Ltd, Dorchester, Dorset, UK) with a 0.2 mm active diameter was employed for testing. Its calibrated frequency range spanned 300 kHz to 16 MHz. The hydrophone detected ultrasound pressure variations, converting acoustic energy proportionally into electrical signals. These signals were then amplified by a hydrophone preamplifier and displayed as waveform images and numerical data via the oscilloscope. The recorded signals were converted into pressure values based on frequency-specific sensitivity calibrations.

Mice were anesthetized with 1.5% isoflurane gas and secured in a stereotaxic frame. After the top of the mice head was depilated, the bregma was identified as the reference point for LHb targeting (coordinates: AP −1.70 mm, ML ±0.42 mm, DV −2.60 mm from bregma). The LHb coordinates were mapped on the skull surface using a stereotaxic apparatus. The ultrasound transducer collimator was then rigidly fixed to the stereotaxic frame and precisely aligned with the left LHb projection point. Ultrasonic coupling gel was applied to ensure complete bubble-free contact between the transducer and skull surface, achieving optimal acoustic energy transmission to the targeted LHb region.

Concurrently, mice in the CRS + Ket-NDs + Sham and CRS + Ket-NDs + US groups received a 0.2 mL tail vein injection of Ket-NDs. Mice in other groups received tail vein injections of 0.2 mL of saline. The ultrasound stimulation parameters were as follows: frequency: 2 MHz, duty cycle: 5%, pulse duration: 500 μs, pulse repetition period: 1 ms, stimulus duration: 300 ms, inter-stimulus interval: 3 s, PNP: 3.08 MPa, treatment regimen: 15 min/day for 3 consecutive days. Sham Control: The transducer was positioned identically without activating the signal generator. All groups received the same gas anesthesia on treatment days. All other conditions remained unchanged.

### 2.12. Evaluation of Antidepressant Efficacy

Following completion of the full treatment protocol, behavioral assessments including SPT, OFT, TST and FST were conducted to evaluate treatment-induced behavioral modifications.

### 2.13. Biosafety Assessment

Following behavioral testing, mice were euthanized for histopathological analysis. Brain tissues: H&E staining was performed to examine pathological alterations in the LHb and surrounding regions (e.g., hemorrhage, neuronal vacuolation). Major organs (heart, liver, spleen, lungs, kidneys): H&E staining was conducted to assess systemic toxicity.

### 2.14. Liquid Chromatography–Tandem Mass Spectrometry (LC-MS/MS) Analysis

Following behavioral tests, mice were sacrificed, and brains were immediately extracted and placed on ice. The LHb was rapidly dissected out with reference to a mice brain stereotaxic atlas. The isolated tissue was rinsed with pre-chilled physiological saline and the surface moisture was removed using filter paper. The tissue was weighed and homogenized in ice-cold 0.1% formic acid aqueous solution at a weight-to-volume ratio of 1 mg:10 μL. Two steel beads were added to each tube, and samples were thoroughly homogenized using a tissue homogenizer. The homogenates were then centrifuged at 13,200× *g* for 20 min at 4 °C. The supernatant was collected, and its volume was recorded. A three-fold volume of 1% formic acid in water–methanol solution (1:1, *v*/*v*) was added to the supernatant. After vigorous vortex mixing, the mixture was centrifuged again under the same conditions (13,200× *g*, 20 min, 4 °C). The final supernatant was collected and stored at −20 °C until LC-MS/MS analysis.

Standard stock solutions of glutamate (Glu) were prepared by accurately weighing 1 mg of each standard and dissolving it in 1% formic acid in water–methanol solution (1:1, *v*/*v*) to a final concentration of 1 mg/mL. Appropriate standard stock solutions were then transferred to volumetric flasks and diluted with 1% formic acid in water–methanol (1:1 *v*/*v*) to prepare mixed standard solutions. These mixed standard solutions were subsequently serially diluted with the solvent to generate calibration solutions of different concentrations for establishing the standard curve.

Chromatographic separation was performed on a Waters Acquity UPLC system (Waters Corp., Milford, MA, USA), equipped with a binary solvent manager, an online degasser, and an autosampler. The system was controlled using MassLynx™ software (version 4.1, Waters Corp.) for data acquisition and processing. Analyte separation was achieved on a reversed-phase column (Waters BEH HILIC C18, 2.1 mm × 100 mm, 1.7 μm) maintained at 30 °C. The mobile phase consisted of acetonitrile and 0.1% aqueous formic acid (65:35, *v*/*v*), delivered isocratically at a flow rate of 0.3 mL/min. The injection volume was 2 μL, and the temperature of the autosampler tray was set at 5 °C.

Detection was carried out using a triple quadrupole mass spectrometer (Waters Corp.) equipped with an electrospray ionization (ESI) source operating in positive ion mode. The ESI source parameters were set as follows: capillary voltage, 3.2 kV; cone voltage, 30 V; desolvation gas flow, 550 L/h; desolvation temperature, 350 °C; and cone gas flow, 150 L/h. The samples were diluted 1000-fold prior to analysis. Quantification was performed via multiple reaction monitoring (MRM). The specific MRM transitions, along with the optimized cone voltages and collision energies, were as follows:

Glu: Precursor ion [M + H]+: *m*/*z* 147.96 → product ion: *m*/*z* 83.99; Collision energy: 20 eV; Dwell time: 0.03 s.

### 2.15. Statistical Analysis

Mice were randomly assigned to each treatment group. In all behavioral experiments, the analysis process was performed blinded to treatment groups. Statistical analysis and graphing were performed using GraphPad Prism 10 software. All statistical tests were two-sided, and *p* < 0.05 was considered statistically significant. All values are expressed as mean ± Standard Error of the Mean (SEM). Normality was assessed using the D’Agostino and Pearson omnibus test, and homogeneity of variance was evaluated using the Brown–Forsythe test. One-way ANOVA was conducted, followed by Dunnett’s T3 multiple comparisons test (for unequal variances) or Bonferroni’s test (for equal variances). Additional tests were applied where appropriate, including linear regression.

## 3. Results

### 3.1. Physical Characteristics and Characterization of Ket-NDs

**Figure 4:**
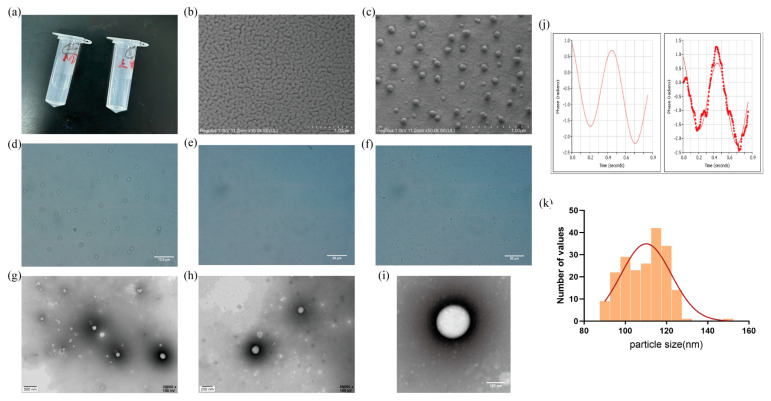
The characterization of Ket-NDs. (**a**) The photographs of the appearance of Ket-NDs and supernatant. (**b**,**c**) Cryo-SEM image of Ket-NDs at different magnifications. (**d**–**f**) Light microscope image of Ket-NDs at different magnifications. (**g**–**i**) TEM image of Ket-NDs at different magnifications. (**j**) The zeta potentials of Ket-NDs. (**k**) The histogram shows the particle size distribution of Ket-NDs, obtained by measuring over 200 individual particles from Cryo-SEM images. The data were fitted to a Gaussian distribution model (red curve).

The prepared Ket-NDs appeared as a semi-transparent white suspension (Figure 4a). Cryo-SEM results demonstrated that Ket-NDs exhibited good dispersibility with spherical morphology and uniform size distribution (Figure 4b,c). Optical microscopy (200× magnification) revealed spherical Ket-NDs with homogeneous size distribution and no obvious aggregation (Figure 4d–f), showing a density of approximately 3.2 × 10^8^ particles/mL. TEM imaging confirmed that Ket-NDs possessed a core–shell structure with regular spherical shape (Figure 4g–i).

Nanoparticle and zeta potential analyzer showed Ket-NDs had an average particle size, as measured by dynamic light scattering (DLS), stabilized at 139.75 ± 9.43 nm (Figure 4k), with a PDI of 0.225 ± 0.025 and zeta potential of −15.93 ± 5.906 mV (Figure 4j). Cryo-SEM analysis of 201 particles yielded a number-average diameter of 109.5 ± 10.4 nm, with percentile values of D_10_: 94.78 nm, D_50_: 110.79 nm, and D_90_: 121.46 nm. The size distribution of Ket-NDs was based on Cryo-SEM image analysis (Figure 4k).

### 3.2. EE and LE

**Figure 5:**
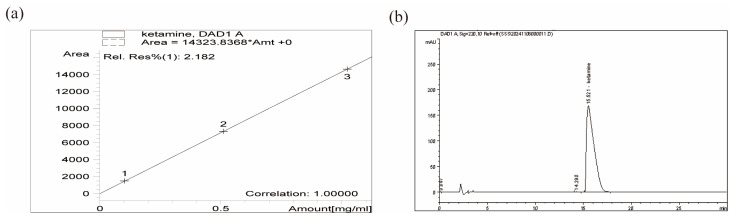
(**a**) Standard calibration curve of Ket: Drug concentration (mg/mL) versus chromatographic peak area. (**b**) RP-HPLC analysis of Ket content in supernatant: Characteristic absorption peak detected at 220 nm.

The standard curve for Ket quantification was established using RP-HPLC by measuring peak areas of Ket standards at various concentrations. The calibration curve demonstrated excellent linearity (R^2^ = 1.00000) between peak area and drug concentration (Figure 5a), indicating a strong linear relationship within the tested concentration range. The regression equation derived from this standard curve was used for subsequent EE and LE calculations.

All supernatants collected during Ket-ND preparation were analyzed by RP-HPLC. Ket content was detected by monitoring the characteristic UV absorption peak at 220 nm. Peak areas were recorded and quantified against the pre-established standard calibration curve to determine the total concentration of free Ket (Figure 5b). Using the EE and LE formulas, the EE of Ket-NDs was determined to be 78.25 ± 16.13%, while the LE was 15.55 ± 4.49%.

### 3.3. Stability Assessment of Ket-NDs

**Figure 6:**
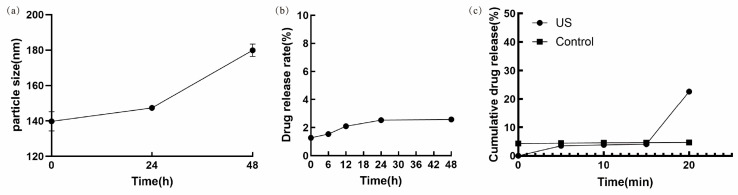
Stability and in vitro release profile of Ket-NDs. (**a**) Change in particle size of Ket-NDs over time under storage conditions at 4 °C. (**b**) Cumulative drug release rate from Ket-NDs over time at 4 °C. (**c**) Comparison of Ket release from Ket-NDs within 20 min at room temperature with and without ultrasound irradiation.

Under 4 °C storage conditions, the particle size of Ket-NDs was 147.34 ± 2.10 nm at 24 h and 179.88 ± 6.38 nm at 48 h, while the cumulative drug release remained low and stable, measuring approximately 2.55% at 24 h and 2.61% at 48 h. The temporal changes in particle size and drug release profile are shown in Figure 6a,b, respectively (Figure 6a,b).

### 3.4. In Vitro Release of Ket-NDs Under Ultrasound Irradiation

Under room temperature conditions without ultrasound irradiation, Ket-NDs exhibited a slow drug release profile, with only 4.54% of Ket released within 20 min. In contrast, upon ultrasound stimulation, Ket was rapidly released from the nanodroplets, achieving a cumulative release of approximately 24.47% over the same period (Figure 6c).

### 3.5. Acoustic Field Measurement

**Figure 7:**
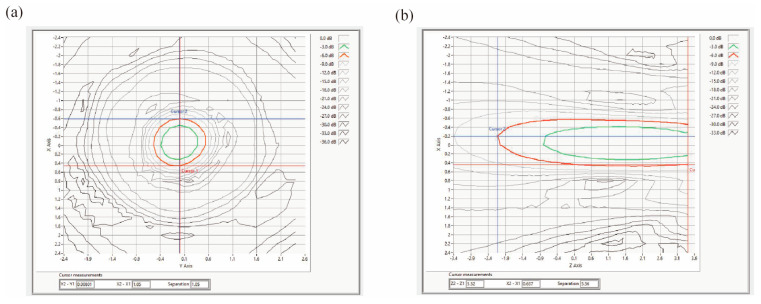
Contour map of axial cross-sectional sound pressure at the focal point of the collimator-equipped probe. (**a**) X-Y plane. (**b**) X-Z plane. The ultrasound emission direction is from Zmax to Zmin.

The Z_MAX_ boundary was located less than 1 mm from the collimator’s acoustic window plane. The focal point was positioned approximately 3 mm from the collimator’s acoustic window plane. At the focus, the −3 dB acoustic pressure beam widths were measured as 0.76 mm in the x-direction, 0.77 mm in the y-direction, and 4.26 mm in the z-direction (Figure 7a,b).

### 3.6. Behavioral Assessments

We investigated whether ultrasound-triggered release of Ket-NDs at LHb target following intravenous administration could produce antidepressant effects. The CRS model, a well-established paradigm for inducing depressive-like behaviors in rodents [42], was employed to validate the therapeutic efficacy of this approach. Our findings demonstrate that this targeted treatment modality effectively improves depression-like phenotypes in the CRS model.

**Figure 8:**
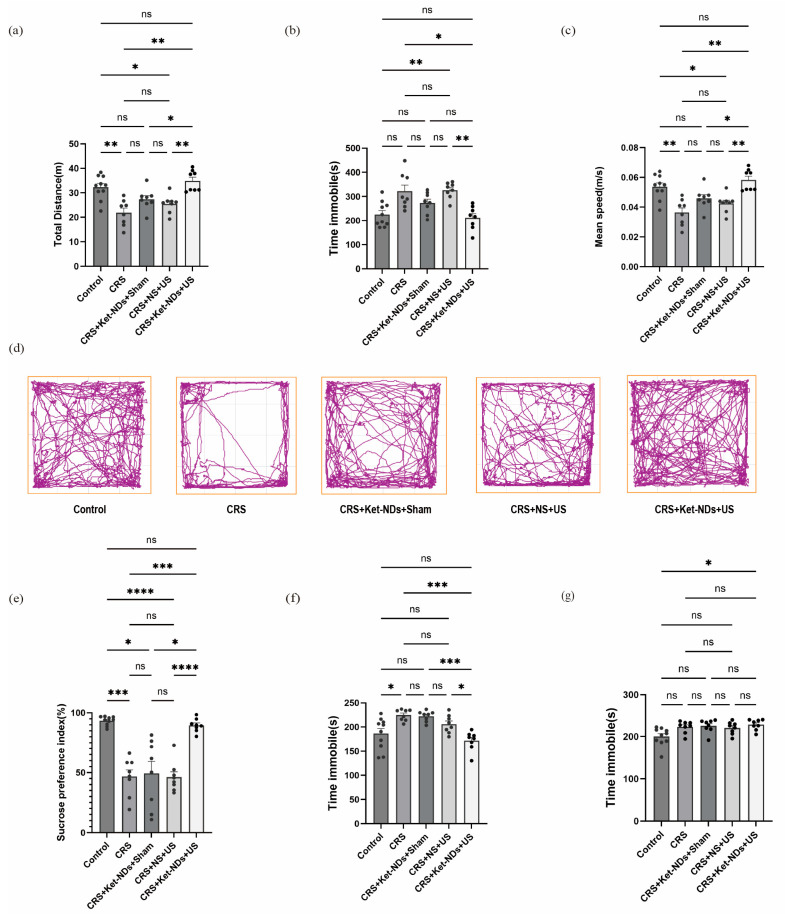
Low-intensity focused ultrasound combined with Ket-NDs improves the behavioral indicators of depression in mice: (**a**–**d**). Open field test, (**a**) moving distance, (**b**) time immobile, (**c**) mean speed, (**d**) trajectory map of each group of mice, (**e**) Sucrose preference test: sucrose preference index, (**f**) Tail Suspension Test: time immobile, (**g**) Forced swim test: time immobile. All data are presented as mean ± SEM (Control: *n* = 10; other groups: *n* = 8 per group). Each dot represents one mice. Statistical analyses were performed using the one-way ANOVA. ns: *p* > 0.05; *: *p* < 0.05; **: *p* < 0.01; ***: *p* < 0.001; ****: *p* < 0.0001.

OFT:

Statistical analysis using Brown–Forsythe ANOVA with Dunnett’s T3 post hoc tests revealed the following. During the 10 min OFT session, mice subjected to CRS exhibited significantly reduced total travel distance compared to control mice (Figure 8a; *p* < 0.01, Mean Diff. = +10.36 m, Control vs. CRS), indicating diminished exploratory behavior. This CRS-induced deficit was fully rescued by Ket-NDs + US treatment (Figure 8a; *p* < 0.01, Mean Diff. = −13.01 m, CRS + Ket-NDs + US vs. CRS), demonstrating restoration of locomotor activity. One-way ANOVA revealed a marginal group effect on immobility time in CRS mice (*p* = 0.0696; Figure 8b). Although the increase in immobility time (Control vs. CRS) did not reach strict significance, Ket-NDs + US treatment significantly reversed this trend (Figure 8b; *p* < 0.05, CRS + Ket-NDs + US vs. CRS). Furthermore, CRS mice showed markedly decreased average movement speed (Figure 8c; *p* < 0.01, Control vs. CRS), which was normalized after Ket-NDs + US intervention (Figure 8c; *p* < 0.01, CRS + Ket-NDs + US vs. CRS). These results collectively indicate that Ket-NDs + US treatment effectively ameliorates CRS-induced locomotor and exploratory deficits, highlighting its potential as an antidepressant therapy.

SPT:

One-way ANOVA with Brown–Forsythe correction (F*(4, 15.08) = 20.13, *p* < 0.0001) revealed significantly lower sucrose preference in CRS mice compared to Controls (Figure 8e; *p* < 0.001, Mean Diff. = 46.66%, Control vs. CRS). Ket-NDs + US treatment completely reversed this anhedonia-like behavior (Figure 8e; Mean Diff. = −42.97%, *p* = 0.0004, CRS + Ket-NDs + US vs. CRS). Notably, the CRS + Ket-NDs + US group showed significantly higher sucrose preference than the CRS + Ket-NDs + Sham and CRS + NS + US groups post-treatment (Figure 8e; *p* < 0.05, CRS + Ket-NDs + US vs. CRS + Ket-NDs + Sham; *p* < 0.0001, CRS + Ket-NDs + US vs. CRS + NS + US).

TST:

CRS mice displayed prolonged immobility duration compared to Controls (Figure 8f; *p* < 0.05, Dunnett’s T3 test, Control vs. CRS), confirming successful induction of behavioral despair. Notably, the CRS + Ket-NDs + US group showed a complete reversal of this effect, with immobility duration significantly reduced versus the CRS group (Figure 8f; *p* < 0.001, CRS + Ket-NDs + US vs. CRS). In contrast, neither US alone nor Ket-ND injection alone showed a statistically significant difference from the CRS group (Figure 8f; *p* > 0.05, CRS + Ket-NDs + Sham vs. CRS; *p* > 0.05, CRS + NS + US vs. CRS), confirming that the antidepressant efficacy depended on targeted Ket release triggered by US.

FST:

Neither chronic restraint stress nor subsequent group therapy significantly altered FST performance (Figure 8g).

### 3.7. Safety and Organ Toxicity Evaluation

**Figure 9:**
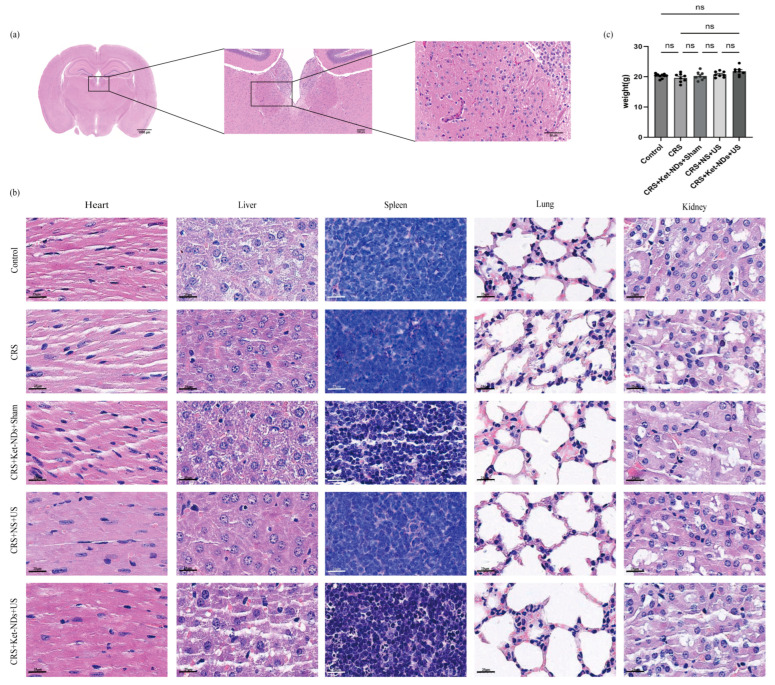
Safety Evaluation: (**a**) H&E staining of brain tissue in mice of the CRS + Ket-NDs + US group post-treatment, revealing no significant hemorrhage or damage in the LHb following ultrasound irradiation. (**b**) Representative H&E images of major organs (heart, liver, spleen, lung, kidney) from all groups showed normal histoarchitecture with no observable pathological changes. (**c**) Body weight comparison across all groups after treatment showed no statistically significant differences. All data are presented as mean ± SEM (Control: *n* = 10; other groups: *n* = 8 per group). Each dot represents one mice. Statistical analyses were performed using the one-way ANOVA. ns: *p* > 0.05.

To assess treatment safety, potential hemorrhage in target brain regions was analyzed using H&E staining. Brain sections from CRS + Ket-NDs + US group mice were fixed on slides, stained, and meticulously examined under microscopy. Notably, H&E analysis demonstrated absence of significant hemorrhage or structural damage in the LHb after ultrasound-triggered targeted delivery of Ket-NDs (Figure 9a), validating the precision safety of this combinational therapeutic approach.

Histopathological evaluation via H&E staining of major organs (heart, liver, spleen, lungs, kidneys) across all experimental groups demonstrated preserved tissue architecture without observable pathological alterations. Key findings included the absence of edema or cellular shrinkage, intact cellular membranes, normal nuclear morphology, and no evidence of chromatin fragmentation or vacuolation (Figure 9b). Critically, the lack of significant lesions in the CRS + Ket-NDs + US group confirms the biosafety profile of Ket-NDs in vital organs. Moreover, the absence of statistically significant differences in body weight between Ket-ND-treated mice and other groups after treatment further suggests the lack of acute systemic toxicity (Figure 9c).

### 3.8. LC-MS/MS Analysis of Neurotransitters

**Figure 10:**
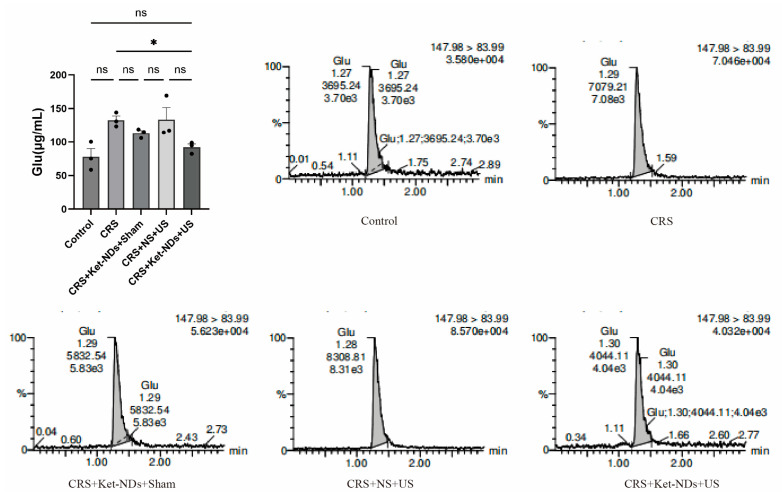
Comparison of glutamate (Glu) levels in the LHb region. The top left panel displays the statistical graph of the area under the curve. The remaining panels show the chromatogram profiles for each group. A decrease was observed in the CRS + Ket-NDs + US group compared to the CRS group (*n* = 3 per group). Each dot represents one mice. All data are presented as mean ± SEM. One-way ANOVA was used for statistical analysis. * *p* < 0.05. ns: *p* > 0.05.

The measurement of glutamate (Glu) levels yielded the following results: Control group = 78.0 ± 12.1 μg/mL, CRS group = 132.8 ± 6.1 μg/mL, CRS + Ket-NDs + Sham group = 113.2 ± 3.8 μg/mL, CRS + NS + US group = 133.5 ± 17.9 μg/mL, and CRS + Ket-NDs + US group = 92.0 ± 5.0 μg/mL. The glutamate level in the CRS + Ket-NDs + US group was significantly lower than that in the CRS group (*p* < 0.05). However, no statistically significant differences were observed compared to the other groups (Figure 10).

## 4. Discussion

Despite ketamine’s rapid antidepressant efficacy, particularly in treatment-resistant depression, its clinical utility is restricted by systemic side effects and abuse potential. Targeted brain delivery offers a promising solution. While MB-enhanced focused ultrasound safely opens the blood–brain barrier (BBB) via cavitation and mechanical effects [43,44], conventional MBs (1–5 µm diameter) cannot efficiently traverse tissue gaps (100–750 nm) and suffer from rapid clearance and poor target accumulation [45].

Phase-change NDs overcome these limitations. Perfluorocarbon-core NDs undergo ADV upon ultrasound exposure, generating cavitation-capable MBs for BBB opening [27]. Their nanoscale size facilitates penetration through the opened BBB, aided by the enhanced permeability and retention (EPR) effect [46]. Transcranial focused ultrasound (tFUS) combined with NDs enables localized neuropharmacological delivery [21,47]. Upon ultrasound irradiation, the NDs vaporize into MBs; the combined use of FUS and MBs significantly reduces the acoustic intensity required for non-invasive blood–brain barrier opening [48]. Furthermore, the ultrasound-mediated BBB opening is reversible, with recovery occurring rapidly within hours [49]. ND-based systems also demonstrate enhanced brain delivery efficiency with reduced endothelial damage versus MBs [50,51,52].

Our strategy encapsulates Ket within ND shells for intravenous administration. Stereotaxic localization of the LHb was performed using standard brain atlases. Subsequent LIFU irradiation of the target region induced ADV of circulating Ket-NDs, generating MBs that underwent instantaneous cavitation. This process created sonoporation effects, forming transient pores in cellular membranes and disrupting tight junctions, thereby achieving noninvasive BBB opening for localized high-concentration Ket release. This approach enhances bioavailability while minimizing systemic exposure.

Common shell materials for preparing Ket-NDs include lipids [53], poly (lactic-co-glycolic acid) (PLGA) [54], surfactants, proteins, etc. We selected biocompatible phospholipids for the shell. The liquid core comprised PFP, a perfluorocarbon chosen for its low phase-transition temperature (29 °C), chemical stability, biocompatibility, biological inertness (enabling pulmonary clearance), and established utility in ultrasound-triggered drug delivery [55]. Using a modified rotary evaporation/emulsification method [42], we optimized parameters to synthesize Ket-NDs with the following key physical characteristics: a small average hydrodynamic diameter of 139.75 ± 9.43 nm, as measured by DLS, negative zeta potential (−15.93 ± 5.906 mV), and PDI of 0.225 ± 0.025, indicating a uniform distribution, and good monodispersity. This negative surface charge minimizes nonspecific protein adsorption, enhancing circulatory stability. The high encapsulation efficiency (EE: 78.25 ± 16.13%) and drug loading capacity demonstrate effective Ket loading, addressing issues of systemic side effects and rapid metabolism, and enabling subsequent targeted delivery.

Complementary Cryo-SEM analysis, which provides high-resolution visualization of the nanoparticle core in a solid state, yielded a number-average diameter of 109.5 ± 10.4 nm (*n* = 201 particles) with a percentile distribution (D_10_: 94.78 nm, D_50_: 110.79 nm, D_90_: 121.46 nm). The observed reduction in nanoparticle diameter measured via Cryo-SEM compared to that obtained by DLS can be attributed to intrinsic methodological differences and the material properties of the Ket-NDs. We posit that the key factor underlying this discrepancy lies in the phase-transition behavior of the PFP core (boiling point: 29 °C). During Cryo-SEM sample preparation, rapid vitrification in liquid nitrogen slush (−210 °C) solidifies the initially liquid PFP core. We hypothesize that this solidification process induces a degree of volumetric contraction, likely due to the increased molecular packing density in the solid state. In contrast, DLS measurements were conducted at 4 °C, a temperature at which PFP remains liquid and thermally expanded. Consequently, Cryo-SEM captures the contracted, frozen core–shell morphology, while DLS reports the hydrodynamic diameter of the swollen, liquid Ket-NDs in suspension.

This study combines transcranial LIFU stimulation technology with a drug-loaded ND system. We employed a custom head-mounted LIFU device (developed collaboratively with SIAT-CAS) specifically designed for mice. Its main components include a small transducer crystal, a concave epoxy acoustic lens, a ring-shaped housing, and external connecting wires leading to the main unit, operating at 2–5 MHz with an acoustic intensity range of 500–2000 kPa. Key features include real-time focusing capability and modular design for optimal impedance and tuning matching, ensuring efficient ultrasound transmission. In previous work, this device combined with MBs improved addiction-related behaviors through nucleus accumbens (NAc) stimulation [29]. In contrast, the current study successfully ameliorated depressive-like behaviors using our self-made Ket-NDs, establishing a comprehensive system from development to preclinical validation.

Our selection of 2 MHz was based on a comprehensive consideration of ADV, MBs-mediated sonoporation, and transcranial penetration requirements. Upon ultrasound irradiation in the target brain region (LHb), Ket-NDs undergo ADV phase transition into MBs, which serve as critical mediators for subsequent sonoporation-enhanced drug delivery. As demonstrated by Wang et al. [56], MBs composed of phospholipids—similar in composition to the ADV-generated MBs in our experiments—exhibit a resonance frequency close to 2 MHz. Operating at this resonant frequency maximizes oscillation amplitude and energy efficiency, thereby enhancing mechanical effects (e.g., microjetting and shear stress) that facilitate effective sonoporation and subsequent Ket delivery. Moreover, ultrasound frequency is inversely related to tissue penetration depth. Given that our study emphasizes targeted drug delivery rather than imaging, penetration depth takes precedence over resolution. A frequency of 2 MHz provides a favorable balance, enabling sufficient penetration to reach brain structures. Although the linear resonance frequency of typical 200–300 nm PFC NDs is considerably higher than 2 MHz, numerous studies demonstrate that ADV can be efficiently triggered off-resonance, primarily depending on PNP and ND concentration [57,58,59]. The pressure used in our study (3.08 MPa) exceeds these reported thresholds, ensuring efficient ADV initiation. Under such pressures, NDs can undergo nonlinear expansion leading to vaporization, and subsequent low-frequency oscillation may further enhance localized shear stress to promote drug release.

Depression presents with diverse and complex symptoms that vary among patients, making it unrealistic to replicate all symptoms in animal models. In practice, an animal model that demonstrates several core symptoms is considered successful [60]. SPT, FST, OFT, and TST are commonly used methods to assess depressive-like behaviors in rodents. In this study, chronic restraint stress (14 days) induced core depressive-like phenotypes in mice: reduced OFT locomotion (diminished exploration), decreased SPT index (anhedonia), and increased TST immobility. These behaviors were significantly improved in the CRS + Ket-NDs + US treatment group. Notably, the Ket dose delivered via a single 0.2 mL tail vein injection of Ket-NDs was substantially lower than the typical effective systemic dose (10 mg/kg i.p.) [4].

A methodological consideration of this study is the fixed order of behavioral testing, which may lead to carry-over effects. Our sequence commenced with the least stressful assays (SPT) and progressed to the most aversive ones (FST). This approach is commonly used in behavioral research [61,62,63] to prevent acute stress from the latter tests from confounding the measurements of basal hedonic state (SPT) and spontaneous anxiety (OFT). However, this design inherently risks that prior exposure to the OFT and TST may modulate behavior in the subsequent FST. While the 24 h intervals between tests were intended to allow for recovery, we cannot rule out the possibility of learned helplessness or stress sensitization influencing the outcomes of the later tests. Therefore, the results from the TST and FST should be interpreted with this potential interaction in mind. Despite this, the consistent and parallel outcomes observed across multiple behavioral domains strengthen the overall conclusions of our study.

The lack of significant changes in FST immobility time may be related to the small sample size. The acute antidepressant effects of Ket typically peak within 24 h after administration. As demonstrated by Hu Hailan’s team, targeted Ket delivery to the rat LHb produced significant improvement in depressive-like behaviors within 1 h [4]. In our study, considering the need to order behavioral tests by stress level (with FST being most stressful), we performed FST last, which may have missed the peak drug effect period. Importantly, TST, which similarly assesses despair-like behavior, showed significant improvement. Therefore, future studies should include detailed time-course experiments to determine the optimal therapeutic time window and duration of the antidepressant effect. Addressing this will be crucial for translating this promising strategy into clinical practice.

Previous studies [64] have utilized focused ultrasound in conjunction with GDNF-loaded MBs to transiently open the BBB and alleviate depression in rats. In our study, the Ket-NDs employed exhibited greater stability and were capable of directly releasing high concentrations of the drug to act on the intracranial target (LHb). Research by Gouveia et al. [37] has demonstrated that anesthetic-loaded NDs targeted to the amygdala can reduce agitation in Alzheimer’s mice. Our approach is similar, targeting a key brain region associated with Ket’s mechanism of action, leveraging LIFU-triggered Ket-NDs for localized drug delivery to avoid systemic drug side effects.

Glu is the most abundant excitatory neurotransmitter in the brain; however, excessive release of Glu can lead to excitotoxic effects. Glutamatergic neurotransmission in the LHb plays a critical role in the pathogenesis of depression [65]. Previous studies have demonstrated that animals with depression-like behaviors exhibit significantly elevated levels of glutamate in the LHb and overactivation of NMDA receptors [66]. In the present study, Glu levels in the CRS group were elevated compared with those in the Control group, although the difference was not statistically significant, potentially due to the limited sample size. Notably, compared with the CRS group, the CRS + Ket-NDs + US group showed a significant reduction in Glu levels, suggesting that targeted release of Ket via ultrasound-triggered nanodroplets may alleviate depressive-like behaviors by attenuating excessive glutamatergic excitation in the LHb. Next, we plan to increase the sample size and incorporate electrophysiological studies to further elucidate the underlying mechanisms.

It should be noted that the cerebral cortex along the ultrasound path to the LHb was inevitably irradiated. However, owing to the highly focused nature of the LIFU employed, the acoustic energy outside the target region decays rapidly and remains well below the threshold for inducing structural damage or significant off-target neuromodulatory effects, as visually corroborated by the acoustic field distribution provided in Figure 7. Although thermal, cavitational, and mechanical effects could theoretically occur within the focal zone, the selected ultrasound parameters and precise stereotactic targeting minimized such risks. Histological results showed no obvious tissue damage or hemorrhage in both target and surrounding areas of mice receiving 15 min daily treatments for 3 consecutive days. Furthermore, long-term safety can also be partially assessed through potential motor impairments. We observed no differences in locomotor activity (OFT) and TST performance between CRS + Ket-NDs + US and control groups, indicating the basic safety of our LIFU approach.

In summary, we developed stable, high-EE Ket-NDs. Combined with targeted transcranial LIFU, this system achieves non-invasive, spatiotemporally controlled drug delivery via cavitation-induced BBB opening and localized droplet vaporization. This strategy enables high-concentration Ket release specifically within the LHb, producing significant antidepressant effects at low systemic doses while enhancing bioavailability and minimizing side effects and addiction risk. It offers a novel precision approach for depression treatment and a paradigm for CNS-targeted therapy. While behavioral changes confirm successful delivery, quantification of neurotransmitter dynamics, electrical activity (particularly LHb burst firing suppression), and detailed mechanism exploration are needed. As a feasibility study, only a 3-day treatment regimen was evaluated; subsequent work should determine the minimum effective dose and frequency. Prior to clinical translation, further optimization of ultrasound parameters and comprehensive long-term histological/ultrastructural safety assessments are essential.

## 5. Conclusions

In this study, we successfully developed Ket-NDs with favorable physicochemical properties, including uniform distribution, small nanoparticle diameter, good stability, and negative surface charge. By combining Ket-NDs with transcranial LIFU targeted to the LHb, we achieved efficient and specific delivery of low-dose Ket to the desired brain region. This approach significantly ameliorated depression-like behaviors in mice while demonstrating excellent biosafety with no observable toxicity in major organs. Furthermore, this method enables precise regional targeting and localized drug accumulation, thereby enhancing the therapeutic efficacy of Ket and addressing the limitations of systemic administration by minimizing off-target effects. Future studies will focus on further optimizing the ultrasound parameters to improve the drug release efficiency of Ket-NDs and maximize their therapeutic potential, along with conducting long-term safety studies to facilitate clinical translation. Our findings provide a novel therapeutic strategy for the treatment of depression.

## Data Availability

The original contributions presented in this study are included in the article; further inquiries can be directed to the corresponding author.

## Abbreviations

The following abbreviations are used in this manuscript:

ADV	Acoustic droplet vaporization
BBB	Blood–brain barrier
CED	Convection-enhanced delivery
CRS	Chronic restraint stress
CSF	Cerebrospinal fluid
Cryo-SEM	Cryogenic Scanning Electron Microscopy
DPPC	1,2-dipalmitoyl-sn-glycero-3-phosphatidylcholine
DPPG	1,2-Dipalmitoyl-sn-glycero-3-phospho-(1′-rac-glycerol)
DSL	Dynamic light scattering
DSPE	1,2-Distearoyl-sn-glycero-3-phosphoethanolamine
EE	Encapsulation efficiency
EPR	Enhanced permeability and retention
FST	Forced swim test
Glu	Glutamate
H&E	Hematoxylin and eosin
IV	Intravenous
Ket	Ketamine
Ket-NDs	Ketamine-loaded nanodroplets
LE	Loading efficiency
LHb	Lateral habenula
LIFU	Low-intensity focused ultrasound
MBs	Microbubbles
NAc	Nucleus accumbens
NDDS	Nano-drug delivery systems
NDs	Nanodroplets
NMDA	N-methyl-D-aspartate
NS	Normal saline
OFT	Open field test
PBS	Phosphate buffered saline
PDI	Polydispersity index
PFP	Perfluoropentane
RP-HPLC	Reversed-Phase High Performance Liquid Chromatography
SEM	Standard Error of the Mean
SIAT-CAS	Shenzhen Institutes of Advanced Technology, Chinese Academy of Sciences
SPT	Sucrose preference test
TEM	Transmission electron microscopy
tFUS	Transcranial focused ultrasound
TST	Tail suspension test
T-VSCC	T-type voltage-sensitive calcium channels
WHO	World Health Organization
